# “Reverse” Dual Stimulation Has Comparable Efficacy, but Higher Efficiency, than Two Conventional Follicular Phase Stimulations in Poor Responders Undergoing In Vitro Fertilization

**DOI:** 10.3390/jcm15020582

**Published:** 2026-01-11

**Authors:** Andrea Roberto Carosso, Chiara Benedetto, Bernadette Evangelisti, Marco Carosso, Gianvito Contangelo, Stefano Canosa, Gianluca Gennarelli, Alberto Revelli

**Affiliations:** 1 Division of Gynecology and Obstetrics 1, Department of Surgical Sciences, City of Health and Science, University of Turin, 10126 Turin, Italy; acarosso@cittadellasalute.to.it (A.R.C.);; 2 IVIRMA, Global Research Alliance, LIVET, 10126 Turin, Italy; 3 Division of Gynecology and Obstetrics 2, Department of Surgical Sciences, City of Health and Science, University of Turin, 10126 Turin, Italy

**Keywords:** DuoStim, poor responder, Bologna criteria, luteal phase stimulation, reverse dual stimulation, time to pregnancy

## Abstract

**Background/Objectives**: Dual stimulation starting in the follicular phase allows retrieval of more oocytes than single follicular-phase controlled ovarian stimulation (COS). However, dual stimulation excludes fresh embryo transfer (ET), forcing us to postpone the first ET. If dual stimulation is performed in a reverse way (“reverse”-dual stimulation, R-DS), fresh ET can be performed, potentially reducing the time to pregnancy. The aim of the present study is to investigate reproductive outcomes of R-DS compared to two consecutive COS starting in the follicular phase (2FP-COS). **Methods**: A retrospective study was performed on 146 poor responders matching Bologna criteria, among which 45 underwent R-DS and 101 received 2FP-COS. In the R-DS group, the first COS began 5 days after ovulation and the second 5 days after oocyte retrieval. The primary outcome was the time to pregnancy. **Results**: In R-DS, stimulation length, retrieved oocytes, and blastocyst formation rate were comparable in the luteal and follicular COS rounds. Circulating progesterone was always <1.0 ng/mL at ovulation trigger, and fresh ET was performed with a mean endometrial thickness of 9.27 ± 2.28 mm. Comparing R-DS and 2FP-COS, no differences were found in terms of retrieved oocytes and cumulative live birth rate; however, the R-DS group showed significantly shorter time to pregnancy (52.9 ± 11.6 vs. 103.2 ± 23.2 days, *p* < 0.05). **Conclusions**: This study suggests that R-DS is not inferior to two consecutive COS starting in the follicular phase in terms of oocytes retrieved and cumulative live birth rate. R-DS allows immediate fresh ET and can significantly shorten the time to pregnancy, a relevant issue for poor responders’ patients.

## 1. Introduction

It is widely recognized that the outcome of in vitro fertilization (IVF) is influenced by a complex interplay of multiple factors; among these, the total number of oocytes retrieved during ovarian stimulation appears to be one of the most critical determinants, as it shows a strong and direct association with the cumulative live birth rate [1]. Primarily as a consequence of socio-economic factors, including delayed childbearing related to education, career development, and financial considerations, the average age of women seeking and undergoing in vitro fertilization (IVF) treatment has been steadily and progressively increasing over time; as both the number and quality of oocytes decline with age, the proportion of IVF patients that match the definition of “poor responder” [2] is rising accordingly [3]. Unfortunately, in the context of IVF there is nothing proven to significantly improve the prognosis of poor responders [4]. More than three decades have elapsed since the first efforts were made to define poor ovarian responders (POR); nevertheless, despite extensive research in reproductive medicine, an optimal and universally effective therapeutic strategy for this group of patients has yet to be identified [4]. Given the clinical challenge posed by poor responders, understanding the underlying ovarian physiology—specifically the dynamics of follicular development throughout the menstrual cycle—becomes essential to optimize stimulation strategies. Multiple major and minor waves of follicular development have been extensively documented in a variety of domestic species, including primates. During major follicular waves, a single dominant follicle typically emerges and undergoes selection, whereas minor waves are characterized by the absence of dominant follicle selection and do not generally lead to ovulation. Historically, the traditional “propitious moment” theory proposed that in humans, only a single major wave of follicular development occurs during the intraovulatory period [5].

In a landmark series of studies conducted in 2003, Baerwald and colleagues reported that healthy women exhibit two to three distinct waves of follicular development throughout the intraovulatory period. This observation has opened new directions for the design of ovarian stimulation protocols, challenging previous assumptions about the temporal limitations of follicular recruitment and suggesting novel opportunities for optimizing fertility treatments [6,7].

An innovative therapeutic approach aimed at maximizing oocyte yield in these patients is represented by the so-called “dual stimulation” strategy, which has been developed to enhance ovarian response by performing two consecutive stimulation phases [6,8]. Indeed, it is possible to obtain oocytes of comparable quality by stimulating the ovary both in the follicular and luteal phases of the same cycle [5,9,10], obtaining a higher number of oocytes after dual stimulation compared to a single follicular-phase controlled ovarian stimulation (COS) [11]. Smaller follicles are possibly sensitized during COS, and an increased availability of follicles able to respond to the second luteal phase stimulation has been reported [12].

Dual stimulation represents an interesting approach for patients classified as poor responders; however, a notable limitation of this strategy is that it necessitates postponing embryo transfer (ET) to the subsequent menstrual cycle, requiring the cryopreservation of all retrieved oocytes and/or embryos to allow for optimal timing and endometrial preparation. A novel strategy of dual stimulation consists of performing it in a “reverse” way: first during the luteal phase and then in the incoming follicular phase. This “reverse”-dual stimulation (R-DS) allows us to perform ET with fresh embryo(s) in the same menstrual cycle of the second COS, potentially shortening the time to pregnancy. In the present study, we aimed to compare the results obtained by R-DS with those of a conventional COS performed in the follicular phase in two different consecutive months (2FP-COS). Primary outcome of the study was time to pregnancy (TTP), whereas other efficacy parameters (e.g., number of retrieved oocytes, cumulative live birth rate, etc.) were considered secondary outcomes.

## 2. Methods

### 2.1. Patients and Study Design

This study was conducted retrospectively and involved a total of 146 patients who underwent in vitro fertilization (IVF) procedures at the IVF Unit of S. Anna Academic Hospital over a period spanning from March 2020 to September 2023. This study was performed in accordance with the Helsinki Declaration and with approval of the Institutional Review Board (N. 152/2020 and 0025097/2024). Patients’ consent was obtained according to the Institutional Review Board rules.

All patients included in this study were classified as poor responders according to the Bologna criteria [2], defined by the presence of at least two of the following conditions: advanced maternal age (≥40 years) or other risk factors for poor ovarian response, a previous poor ovarian response (≤3 oocytes retrieved following a conventional ovarian stimulation protocol), and/or abnormal ovarian reserve tests (anti-Müllerian hormone levels < 0.5–1.1 ng/mL and/or antral follicle count < 5–7). All patients underwent embryo transfer(s) at the blastocyst stage.

Among the included women, 45 underwent reverse dual stimulation (R-DS) and 101 the conventional approach, with two follicular-phase COS in different months (2FP-COS). The choice of stimulation strategy was based on clinical judgment and logistical considerations, including the need to reduce time to pregnancy, previous treatment history, ovarian response in prior cycles, and patient preference. Baseline characteristics were comparable between the two groups, with no marked differences observed, suggesting limited influence of potential confounders.

Patients in the 2FP-COS group completed all ETs of fresh and frozen embryos derived from the first COS and then started the second COS, which was performed in the first cycle after the last ET.

### 2.2. “Reverse” Dual Stimulation (R-DS) Treatment

**Figure 1:**
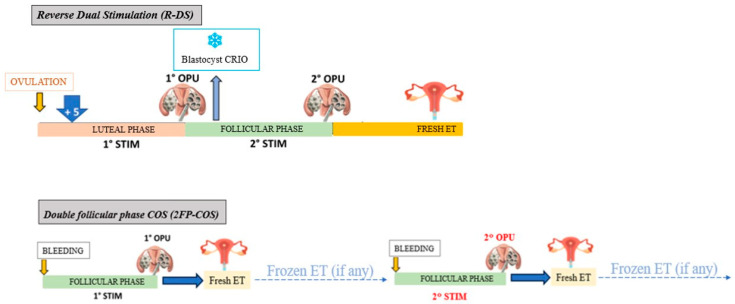
Graphic representation of reverse-dual stimulation (R-DS) and two controlled ovarian stimulation in the follicular phase (2FP-COS). OPU: oocyte pick-up; STIM: ovarian stimulation; CRIO: cryopreservation; ET: embryo transfer.

As described in Figure 1, in the R-DS group the first COS began in the luteal phase, 5 days after ovulation, detected using a urinary LH peak detection kit (Clearblue^®^, Bedford, UK). We did not perform pharmacological luteolysis, according to previous evidence demonstrating comparable oocyte yield with or without previous luteolysis [13]. COS was started with 300 IU/d rFSH + rLH 2:1 ratio (Pergoveris, Merck, Darmstadt, Germany), and follicular growth was monitored by circulating E2 assay and transvaginal US every second day from COS day 6. GnRH-antagonist (Cetrorelix, Cetrotide, Merck-Serono or Ganirelix, Orgalutran, MSD, Rahway, NJ, USA) was administered daily (0.25 mg subcutaneously) from COS day 5. When at least two follicles reached 17 mm mean diameter, a bolus of 0.2 mg Triptorelin Acetate (Fertipeptyl, Ferring, Germany) was administered subcutaneously to trigger ovulation. US-guided oocyte pick-up (OPU) was performed 36 h later, under local anesthesia (paracervical block).

The aspirated follicular fluids were immediately observed under a stereomicroscope. Cumulus–oocyte complexes (COCs) were washed in buffered medium (Gamete medium, Vitrolife, Sweden), and within 2 h from OPU, oocytes and cumulus cells were separated by gentle pipetting in 600 µL buffered medium containing hyaluronidase (HYASE-10X, Vitrolife, Sweden). Semen samples were examined to assess sperm concentration, motility, and morphology according to the World Health Organization guidelines. Sperm samples were then prepared by density gradient centrifugation in order to select motile, morphologically normal spermatozoa. ICSI was performed on all available oocytes within 4 h after oocyte collection and, after 16–18 h of incubation in a controlled atmosphere, the occurrence of normal fertilization was assessed. Normally fertilized oocytes (zygotes) were cultivated in pre-equilibrated Cleavage medium (Cook, Ireland) overlaid with mineral oil up to day 3 of development; at this stage, a change in medium was performed using a stage-specific medium (Blastocyst medium, Cook, Ireland) until the blastocyst stage. As usual practice in our IVF lab, embryo morphological assessment was performed evaluating embryo morphology on day 2 using the Integrated Morphology Cleavage Score (IMCS) [14] and on day 5 according to The Istanbul Consensus Workshop [15]. All cleaved embryos were kept in culture until the blastocyst stage and vitrified to later be transferred in the uterus, as an immediate transfer would not have been possible due to endometrial characteristics in the luteal phase (high risk of implantation failure or ectopic pregnancy).

The second COS began 5 days after OPU with the same protocol and continued until at least two follicles reached 17 mm in mean diameter, when ovulation was triggered by injecting subcutaneously 10,000 international units (IU) of hCG (Gonasi HP, IBSA, Lugano, Switzerland). Circulating progesterone was measured on the day of the second COS trigger to verify the feasibility of fresh ET. OPU, ICSI, and embryo culture were performed as described.

A fresh, single blastocyst was transferred using the US-guided ET technique described elsewhere [16]. Vaginal luteal support was performed administering 400 mg/d natural progesterone (Progeffik, Effik, Milano, Italy) from the day following OPU. Pregnancy was assessed by serum hCG assay 15 days after ET and then confirmed if at least one gestational sac was visualized on transvaginal US after two further weeks.

### 2.3. Double Follicular-Phase COS (2FP-COS) Treatment

In the 2FP-COS group, the first COS was performed with 300 IU/d rFSH + rLH 2:1 ratio (Pergoveris, Merck, Darmstadt, Germany). GnRH antagonist (cetrorelix, Cetrotide, Merck-Serono; ganirelix, Orgalutran, MSD) was administered daily (0.25 mg subcutaneously) from stimulation day 5. COS continued until at least two follicles reached 17 mm mean diameter, when ovulation was triggered by injecting subcutaneously 10,000 international units (IU) of hCG (Gonasi HP, IBSA, Lugano, Switzerland). ICSI was performed, and a fresh ET was planned with an adequate vaginal luteal support, consisting of 400 mg of natural progesterone (Progeffik, Effik, Milano, Italy). Supernumerary blastocysts were cryopreserved and consecutively transferred before performing the second COS. ET with frozen/thawed blastocysts was performed during natural cycle. The second COS was carried out similarly to the previous one. A representative scheme of 2FP-COS compared with R-DS is shown in Figure 1.

### 2.4. Statistical Analysis

The primary endpoint of the study was the efficiency indicator time to pregnancy, calculated as the time between the starting day of the first COS and the day of the hCG-positive result. Secondary outcomes were efficacy indicators such as the number of retrieved oocytes, blastulation rate, and cumulative live birth rate (CLBR), defined as the proportion of deliveries with at least one live birth per started cycle, including all fresh and/or frozen embryo transfers until one delivery with a live birth or until all embryos were used, whichever occurs first [17]. The power analysis was carried out using SAS^®^ Statistics software v9.4. We considered a significant difference between the two groups of 50% (means 50 vs. 100, standard deviation 20); the power for the primary outcome, time to pregnancy, was 99% given the actual sample size. For the other outcomes, the current sample size provides an overall study power of 82%.

The analysis was performed comparing the two groups (R-DS vs. 2FP-COS). After assessment of normal data distribution with the Shapiro–Wilk test, the comparison between groups was performed using the GraphPad Prism V7 software, applying Student’s parametric *t*-test, the non-parametric Wilcoxon test, as appropriate. Continuous variables were expressed as mean ± standard deviation (SD), whereas categorical variables were expressed as absolute values and percentages. All statistical tests were two-tailed, and a *p* value ≤ 0.05 was considered statistically significant.

## 3. Results

The baseline clinical characteristics and the treatment outcome in the two patients’ groups are shown in Table 1.

No significant differences were observed regarding the demographic data or the main variables related to COS. Of note, the total number of retrieved oocytes, the total number of available blastocysts, the overall duration of ovarian stimulation, and the total FSH consumption were similar between the two groups (Table 1).

Table 2 shows the comparison between the first and second COS rounds in the RDS group.

Endometrial thickness was significantly different in the two COS rounds, and as expected, the endometrial pattern was different: hyperechoic-secretory (type 3) in the luteal phase, and trilinear-proliferative (type 1) in the follicular phase (Table 2). In all patients, circulating progesterone was <1.0 ng/mL at ovulation trigger in the follicular phase COS, and fresh ET could always be performed. No patient dropped out after the first COS, and all completed the R-DS protocol.

Comparing the overall efficacy and efficiency of R-DS vs. 2FP-COS, we observed comparable outcomes as for cumulative pregnancy rate and cumulative live birth rate. In the R-DS group, 66.7% (8/12) and 33.4% (4/12) of pregnancies were obtained after fresh ET and frozen ET, respectively. However, a significantly shorter time to pregnancy was observed in the R-DS group (52.9 ± 11.6 vs. 103.2 ± 23.2 days, *p* < 0.05) (Table 1).

## 4. Discussion

Despite significant technical and methodological advances in assisted reproductive technologies, patients classified as poor responders continue to represent a considerable challenge for specialists in reproductive medicine, due to their consistently lower response rates and the associated difficulties in achieving successful clinical outcomes. Indeed, a disappointing cumulative live birth rate (about 10%) was previously reported by several groups [18,19]. Previous studies showed that repeating IVF up to three cycles might increase the overall cumulative live birth rate [20]. However, it is not rare that some of the patients that experienced failure at the first IVF subsequently drop out of the treatment program: indeed, patients of advanced reproductive age—quite frequent among poor responders—are often afraid of wasting time, and when the time-to-pregnancy becomes long they lose trust in the IVF program, turning towards egg donation or even abandoning the idea of parenthood. Thus, the dropout rate is particularly relevant in case of poor responders and significantly affects results [21,22].

The rate of treatment discontinuation is notably higher among couples with an unfavorable prognosis, such as those characterized by advanced maternal age or by a low yield—or complete absence—of usable embryos in a prior treatment cycle. Additional adverse outcomes in previous cycles, including miscarriage, further increase the likelihood of treatment dropout. Moreover, the psychological burden associated with fertility treatment itself represents a significant contributor to disengagement from care. Specific phases of the IVF process are particularly stress-inducing, including the waiting period for fertilization outcomes and pregnancy test results, the period following unsuccessful treatment attempts, and the interval between consecutive treatment cycles. Consequently, patients with a poor reproductive prognosis or those who have experienced significant psychological strain related to previous treatments often discontinue further IVF interventions [23].

In response to these challenges, recent advances in reproductive medicine over the past decade have sought to improve outcomes for poor responders. Over the past ten years, significant progress has been made in understanding the highly dynamic nature of folliculogenesis, coupled with substantial technological and methodological innovations in in vitro fertilization (IVF) laboratories, including extended blastocyst culture techniques, pre-implantation genetic testing for aneuploidy, and advanced vitrification protocols. These scientific and technological advancements have, in turn, facilitated the design and implementation of novel, non-conventional ovarian stimulation strategies. Collectively, these developments have substantially improved the capacity to manage challenging patient populations, such as those classified as poor ovarian responders, offering new opportunities to optimize oocyte yield and clinical outcomes in this traditionally difficult-to-treat group. To enhance the success of in vitro fertilization (IVF) in patients identified as poor ovarian responders, several authors have suggested the implementation of a dual-stimulation protocol. Evidence from these studies indicates that conducting two consecutive controlled ovarian stimulation (COS) cycles within the same menstrual cycle may lead to an increase in cumulative live birth rates while simultaneously reducing the likelihood of patient dropout from treatment [24]. They clearly showed that, due to multiple follicular maturation waves that occur in the same menstrual cycle, COS can be performed both in the follicular and in the luteal phases of the same month, increasing the number of retrieved oocytes without compromising their quality, which is similar in the two-cycle phases [25]. However, a notable limitation of conventional dual stimulation is that it requires cryopreservation of all embryos, thereby postponing the first embryo transfer (ET).

The idea of performing dual stimulation in a “reverse” way (R-DS), starting the first COS in the luteal phase and continuing with the second COS in the follicular phase of the incoming cycle, is aimed at keeping the possibility of an immediate fresh ET, which is potentially able to relevantly shorten time to pregnancy. The present study retrospectively compared the novel approach named “reverse” dual stimulation (R-DS) with two consecutive follicular phase COS (2FP-COS) in a selected population of poor responders matching Bologna criteria [2].

In the present study, R-DS and 2FP-COS showed comparable results in terms of oocyte yield, blastocyst availability, and cumulative pregnancy and live birth rates. A shorter mean time to pregnancy was observed in the R-DS group. This finding appears to be related to the design of the therapeutic protocol, as the reduction in time to pregnancy largely reflects the structural characteristics of the R-DS strategy, particularly the possibility of performing a fresh embryo transfer. So far, no data are available on R-DS in terms of retrieved oocytes, cumulative live birth rate, and time-to-pregnancy, nor was this novel strategy ever compared with two consecutive COS starting in the follicular phase (2FP-COS). A recent randomized trial compared two COS performed in the same menstrual cycle (conventional dual stimulation) with two COS performed in different menstrual cycles (2FP-COS) and showed a comparable oocyte yield; unfortunately, this trial was underpowered to investigate the impact of the two regimens in terms of live birth rate and time to pregnancy [26]. Other studies using conventional dual stimulation in a population of poor responders obtained a cumulative live birth rate similar to the one we observed: 15% [24] or 17.9% [26] vs. 20% in our R-DS group. Interestingly enough, the R-DS group obtained a higher oocyte yield, 8.3 ± 4.2 vs. 5.5 ± 1.9 [24] and 5.0 ± 3.4 [26], possibly explaining the slight higher cumulative live birth rate in our group. Previous evidence reports that luteal-phase stimulation seems to contribute to conventional stimulation with more oocytes with comparable competence to follicular-phase stimulation, and this data could be explained by the potential positive effect of the first stimulation on the second through an enhancement of follicular recruitment [8]. We are not surprised to have found no differences between the two rounds of R-DS in terms of oocytes retrieved, since both COS in the R-DS setting are performed after a wave of follicular recruitment (in the first luteal phase stimulation, this is physiological after endogenous FSH pulse). This approach may hypothetically have a positive impact on oocytes competence; however, future targeted studies aimed at evaluating oocyte quality comparing R-DS with conventional DS need to be designed.

**Figure 2:**
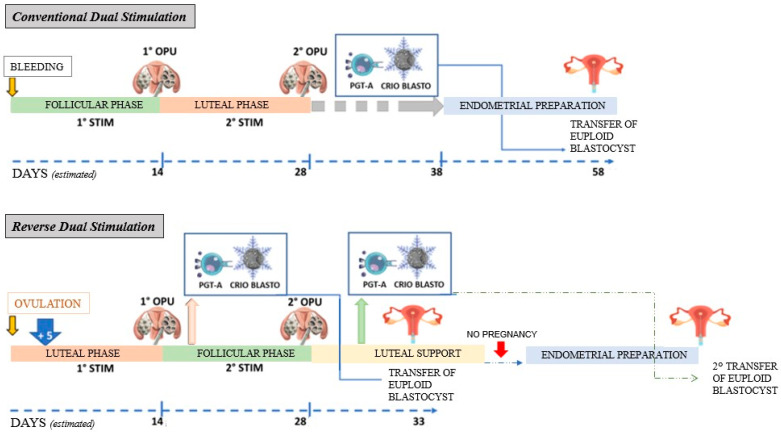
Graphic representation of conventional dual stimulation and reverse-dual stimulation (R-DS) combined with pre-implantation genetic analysis for aneuploidies (PGT-A). OPU: oocyte pick-up; STIM: ovarian stimulation; CRIO: cryopreservation; ET: embryo transfer.

In comparison with conventional dual stimulation, R-DS demonstrates a shorter time to the first ET, which can be performed with a fresh blastocyst. One could speculate that R-DS cannot be combined with a policy of pre-implantation genetic diagnosis for aneuploidy (PGT-a), usually combined with conventional dual-stimulation protocols [24]. However, the proposed algorithm could be particularly useful in IVF centers where PGT-a is not routinely available, or in patients who decline PGT-a for personal, ethical, or economic reasons, with the potential to optimize time to pregnancy. Indeed, the most intuitive approach in the context of R-DS is to proceed with the transfer of a single fresh blastocyst obtained from follicular-ovarian stimulation, leaving the cryopreserved blastocysts obtained from luteal-ovarian stimulation (if any) for subsequent attempts. When PGT-a is indicated, it would still be possible to transfer in utero a cryopreserved euploid blastocyst obtained from luteal-ovarian stimulation onto the endometrium resulting from follicular-ovarian stimulation, leaving any blastocysts obtained in this phase for PGT-a. This approach is summarized in Figure 2.

Of course, this strategy can also be used in the absence of PGT-a, especially if the second stimulation does not result in the formation of a transferable blastocyst. As the endometrium is ready, thawing and transferring blastocysts obtained from the luteal phase may help reduce the time to pregnancy. Further studies will be useful to explore and confirm the potential benefits of this approach.

We are aware that the findings of this study should be interpreted with caution, in light of some methodological limitations. In particular, the retrospective design of the study. In addition, treatment allocation was based on routine clinical practice, including physician judgment, prior treatment characteristics, and patient-related factors. For these reasons, future prospective and randomized studies will be required to confirm these observations.

Although the application of the Bologna criteria, which we applied, is consistent with international consensus, it is well recognized that this definition encompasses a clinically heterogeneous population. In this setting, more recent low-prognosis stratification models such as the POSEIDON classification may provide additional clinical insight. The POSEIDON classification stratifies patients according to age (<35 or ≥35 years), ovarian reserve biomarkers (AMH and antral follicle count), and ovarian response in previous stimulation cycles, identifying four distinct groups with different expected oocyte yields and probabilities of achieving a live birth. Women fulfilling the Bologna criteria largely overlap with POSEIDON groups 3 and 4, which are characterized by a reduced ovarian reserve. In these patients, treatment strategies aimed at maximizing the number of oocytes retrieved within the shortest possible timeframe represent a key therapeutic objective, making an inverted dual stimulation algorithm such as R-DS particularly appealing [27].

A potential direction for future research would be to evaluate the efficacy of R-DS within the different POSEIDON subgroups, which was not possible in this study due to the limited size of the population.

## 5. Conclusions

In conclusion, this preliminary study suggests that in poor responders matching the Bologna criteria, R-DS is not inferior to two consecutive follicular-phase COS as for cumulative live birth rate, allows immediate fresh ET, and can significantly shorten time to pregnancy, a relevant issue for patients with a shortage of time. If confirmed by randomized prospective trials, the advantage observed herein for R-DS in terms of time to pregnancy could open a novel prospective direction for the treatment of poor responders, especially those in advanced reproductive age.

## Figures and Tables

**Table 1:** Baseline characteristics and treatment outcomes of reverse-dual stimulation (R-DS) and double follicular-phase COS (2FP-COS) groups. ns: not significant.

	R-DS (*n* = 45)	2FP-COS (*n* = 101)	*p*
**Woman’s age (years)**	**38.6 ± 4.4**	**38.5 ± 3.9**	***ns***
**Partner’s age (years)**	**41.3 ± 4.9**	**42.1 ± 5.8**	***ns***
**Previous IVF cycles (n)**	**0.8 ± 1.2**	**1.0 ± 1.6**	***ns***
**BMI (kg/m^2^)**	**21.7 ± 3.6**	**22.3 ± 3.4**	***ns***
**Day 3 FSH (IU/L)**	**9.5 ± 3.0**	**10.1 ± 3.1**	***ns***
**AMH (ng/mL)**	**0.8 ± 0.4**	**1.2 ± 1.1**	***ns***
**Antral follicle count (n)**	**7.02 ± 2.7**	**7.8 ± 4.5**	***ns***
**Duration of COS (days)**	**23.1 ± 3**	**22.4 ± 1.9**	***ns***
**Total FSH consumption (IU)**	**7036.7 ± 1042.8**	**6733.6 ± 669.4**	***ns***
**Retrieved oocytes (n)**	**8.3 ± 4.2**	**8.7 ± 4.7**	***ns***
**Ovarian sensitivity index**	**1.2 ± 0.7**	**1.5 ± 0.7**	***ns***
**Blastocysts (n)**	**1.5 ± 1.5**	**1.9 ± 0.9**	***ns***
**Cumulative pregnancy rate/started treatment %**	**26.6% (12/45)**	**31.7% (32/101)**	***ns***
**Cumulative live birth rate/started treatment %**	**20% (9/45)**	**18.8% (19/101)**	***ns***
**Time to pregnancy (days)**	**52.9 ± 11.6**	**103.2 ± 23.2**	***p <* 0.05**

**Table 2:** Treatment outcomes of luteal controlled ovarian stimulation (COS) and follicular controlled ovarian stimulation (COS) in the reverse-dual stimulation (R-DS) group. ns: not significant.

	R-DS		*p*
	**Luteal COS**	**Follicular COS**	
**Total FSH consumption (IU)**	**3600 ± 579.78**	**3574,14 ± 784.12**	***ns***
**Duration of COS (days)**	**11.81 ± 1.94**	**11.48 ± 1.88**	***ns***
**Endometrial thickness (mm)**	**8.09 ± 2.21**	**9.61 ± 2.52**	***<*0.05**
**Number of retrieved oocytes**	**4.11 ± 2.56**	**4.20 ± 2.64**	***ns***
**Mature oocytes**	**3.25 ± 2.25**	**3.16 ± 2.21**	***ns***
**Ovarian sensitivity index**	**1.28 ± 0.89**	**1.30 ± 0.96**	***ns***
**Number of fertilized oocytes**	**2.41 ± 1.83**	**2.09 ± 1.44**	***ns***
**Fertilization rate (%)**	**66%**	**62%**	***ns***
**Blastocysts**	**0.68 ± 0.91**	**0.73 ± 0.82**	***ns***
**Blastulation rate (%)**	**30%**	**29%**	***ns***

## Data Availability

The datasets used and/or analyzed during the current study are available from the corresponding author on reasonable request.
